# Serial binary interval ratios improve rhythm reproduction

**DOI:** 10.3389/fpsyg.2013.00512

**Published:** 2013-08-06

**Authors:** Xiang Wu, Anders Westanmo, Liang Zhou, Junhao Pan

**Affiliations:** ^1^Department of Psychology, Sun Yat-Sen UniversityGuangzhou, China; ^2^Department of Neurology, University of MinnesotaMinneapolis, MN, USA; ^3^Neurology Service, Minneapolis VA Medical CenterMinneapolis, MN, USA

**Keywords:** music, rhythm, binary, ratio, beat, distribution pattern

## Abstract

Musical rhythm perception is a natural human ability that involves complex cognitive processes. Rhythm refers to the organization of events in time, and musical rhythms have an underlying hierarchical metrical structure. The metrical structure induces the feeling of a beat and the extent to which a rhythm induces the feeling of a beat is referred to as its metrical strength. Binary ratios are the most frequent interval ratio in musical rhythms. Rhythms with hierarchical binary ratios are better discriminated and reproduced than rhythms with hierarchical non-binary ratios. However, it remains unclear whether a superiority of *serial* binary over non-binary ratios in rhythm perception and reproduction exists. In addition, how different types of serial ratios influence the metrical strength of rhythms remains to be elucidated. The present study investigated serial binary vs. non-binary ratios in a reproduction task. Rhythms formed with exclusively binary (1:2:4:8), non-binary integer (1:3:5:6), and non-integer (1:2.3:5.3:6.4) ratios were examined within a constant meter. The results showed that the 1:2:4:8 rhythm type was more accurately reproduced than the 1:3:5:6 and 1:2.3:5.3:6.4 rhythm types, and the 1:2.3:5.3:6.4 rhythm type was more accurately reproduced than the 1:3:5:6 rhythm type. Further analyses showed that reproduction performance was better predicted by the distribution pattern of event occurrences within an inter-beat interval, than by the coincidence of events with beats, or the magnitude and complexity of interval ratios. Whereas rhythm theories and empirical data emphasize the role of the coincidence of events with beats in determining metrical strength and predicting rhythm performance, the present results suggest that rhythm processing may be better understood when the distribution pattern of event occurrences is taken into account. These results provide new insights into the mechanisms underlining musical rhythm perception.

## Introduction

Rhythm refers to the organization of events in time. Most musical rhythms, especially in Western music, have an underlying hierarchical metrical structure with multiple levels of temporal periodicity (Essens, 1986; Palmer and Krumhansl, 1990; Honing, 2002; Large et al., 2002; Patel et al., 2005; Zatorre et al., 2007; Grahn and Rowe, 2009; Iversen et al., 2009). The most salient metrical level is a regular beat marking equally spaced points in time, to which people often synchronize movements (Lerdahl and Jackendoff, 1983; Patel et al., 2005; Repp, 2005; Fitch and Rosenfeld, 2007; Grahn, 2009; Winkler et al., 2009). A higher level of temporal periodicity is usually the meter grouping beats (e.g., a march groups beats by twos, whereas a waltz groups beats by threes), resulting in a periodic alternation of strong and weak beats (Lerdahl and Jackendoff, 1983; Palmer and Krumhansl, 1990; Patel et al., 2005; Fujioka, 2009). The metrical structure of a musical rhythm induces the feeling of a beat and the extent to which a rhythm induces the feeling of a beat is referred to as its metrical strength (Povel and Essens, 1985; Patel et al., 2005; Chen et al., 2008; Grube and Griffiths, 2009). A metrical rhythm refers to a rhythm that can be partitioned into equal time intervals and a non-metrical rhythm refers to a rhythm that cannot be partitioned into equal time intervals (Essens and Povel, 1985; Chen et al., 2008). Strongly or weakly metrical rhythms refer to rhythms that strongly or weakly induce the feeling of a beat, respectively (Patel et al., 2005; Chen et al., 2008; Grube and Griffiths, 2009).

**Figure 1:**
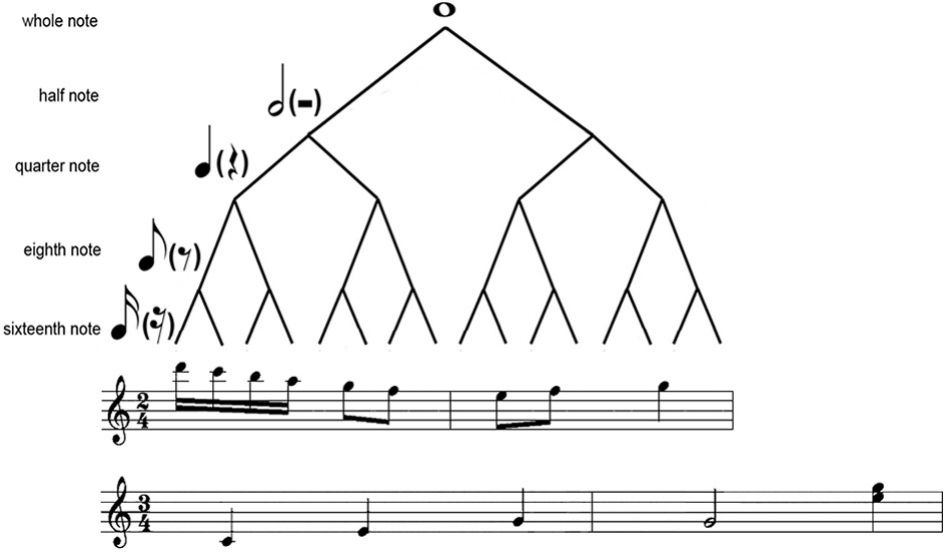
**Illustration of the hierarchical structure.** Shown is a metrical tree (Longuet-Higgins and Lee, 1984; Fitch and Rosenfeld, 2007) with 2 two-measure musical samples (from “Rondo Alla Turca” and “Blue Danube”, respectively) in 4/2 or 3/4 meter.

The periodical metrical levels are organized in a hierarchical structure (Figure 1). This hierarchical structure results in hierarchical (nested) interval ratios, with lower metrical levels nested within higher metrical levels (e.g., 1:2 ratio in a duple meter and 1:3 ratio in a triple meter). Therefore, hierarchical ratios refer to the ratios in theoretical metrical structures, and different meters are characterized by different hierarchical ratios (Repp et al., 2011). A rhythm consists of successive intervals, and the ratios between the successive intervals in a rhythm are here referred to as serial ratios (Repp et al., 2011). A notable feature of musical rhythms is that binary ratios are most frequent at both the hierarchical and serial levels (Fraisse, 1982; Lerdahl and Jackendoff, 1983; Stoffer, 1985), though hierarchical ternary ratios are common in a triple meter.

A relationship between metrical strength and hierarchical binary ratios is suggested by experimental evidence with regard to the improvement of rhythm performance. Rhythm discrimination and reproduction are better for metrical than for non-metrical rhythms (Essens and Povel, 1985; Drake and Gérard, 1989; Chen et al., 2008); better for strongly metrical rhythms than for weakly metrical rhythms (Drake, 1993; Patel et al., 2005; Chen et al., 2008; Grahn and Rowe, 2009). Rhythm discrimination and reproduction are also better for rhythms in a duple meter (or with binary subdivision of a beat) than for rhythms in a triple meter (or with ternary subdivision of a beat) (Rena, 1987; Drake, 1993; Bergeson and Trehub, 2006; Gerry et al., 2010) or a 7/8 Balkan meter (Snyder, 2006). In terms of ratios, these studies (Rena, 1987; Drake, 1993; Bergeson and Trehub, 2006; Snyder, 2006; Gerry et al., 2010) suggest an advantage for hierarchical binary over non-binary ratios. Bergeson and Trehub (2006) and Drake (1993) further found the binary advantage in rhythm discrimination and reproduction when comparing rhythms in 2/4 meter containing only hierarchical binary ratios to rhythms in 3/4 meter containing only hierarchical ternary ratios (one event on each beat, and without events between beats). Rhythms in a binary or ternary meter also commonly contain serial binary and non-binary ratios. The results of Bergeson and Trehub (2006) and Drake (1993) therefore provide further evidence for the advantage of hierarchical binary over non-binary ratios (at least for simple hierarchical ratios), since serial ratios were kept 1:1 in both duple and triple meters in their studies.

Despite the superiority of hierarchical binary over non-binary ratios in rhythm discrimination and reproduction (Rena, 1987; Drake, 1993; Bergeson and Trehub, 2006; Snyder, 2006; Gerry et al., 2010), it is unclear whether a superiority of serial binary over non-binary ratios in rhythm perception and reproduction exists. In fact, studies investigating serial ratios did not observe a consistent binary over non-binary superiority in rhythm perception and reproduction. Essens and Povel (1985), Essens (1986) found no difference between the reproduction of metrical rhythms with binary (1:2) or non-binary integer (1:3) ratios, and both were better reproduced than metrical rhythms with non-integer ratios (1:2.5 or 1:3.5). In addition, non-metrical rhythms with binary ratios (1:2) were better reproduced than non-metrical rhythms with non-binary integer ratios (1:3) (Essens and Povel, 1985). Sakai et al. (1999) constructed rhythms with binary (1:2:4), integer (1:2:3), or non-integer (1:2.5:3.5) ratios. Rhythm duration was varied. They did not find significant differences between the reproduction of rhythms with binary or integer ratios, and both were better reproduced than those with non-integer ratios. In other studies requiring reproduction of sequences with varying ratios, the reproduced ratios showed a tendency toward the 1:2 ratio (Fraisse, 1956; Povel, 1981; Repp et al., 2002), indicating that the 1:2 ratio may be more accurately reproduced than the 1:3 ratio. The deviation in the direction of the 1:2 ratio was also found in a perceptual task (Repp et al., 2011).

The inconsistent observations regarding the superiority of serial binary over non-binary ratios among previous results may be partly related to whether a rhythm is metrical or non-metrical (Essens and Povel, 1985; Drake and Gérard, 1989; Chen et al., 2008). This inconsistency could also be attributable to the fact that some experimental parameters were not kept constant when comparing rhythms with different ratio types. The possible confounding factors include the permutation of time intervals (Essens and Povel, 1985), the number of time intervals (Essens, 1986; Drake, 1993), and the rhythm duration (Essens and Povel, 1985; Sakai et al., 1999). These confounding factors were carefully controlled in the present study.

**Figure 2:**
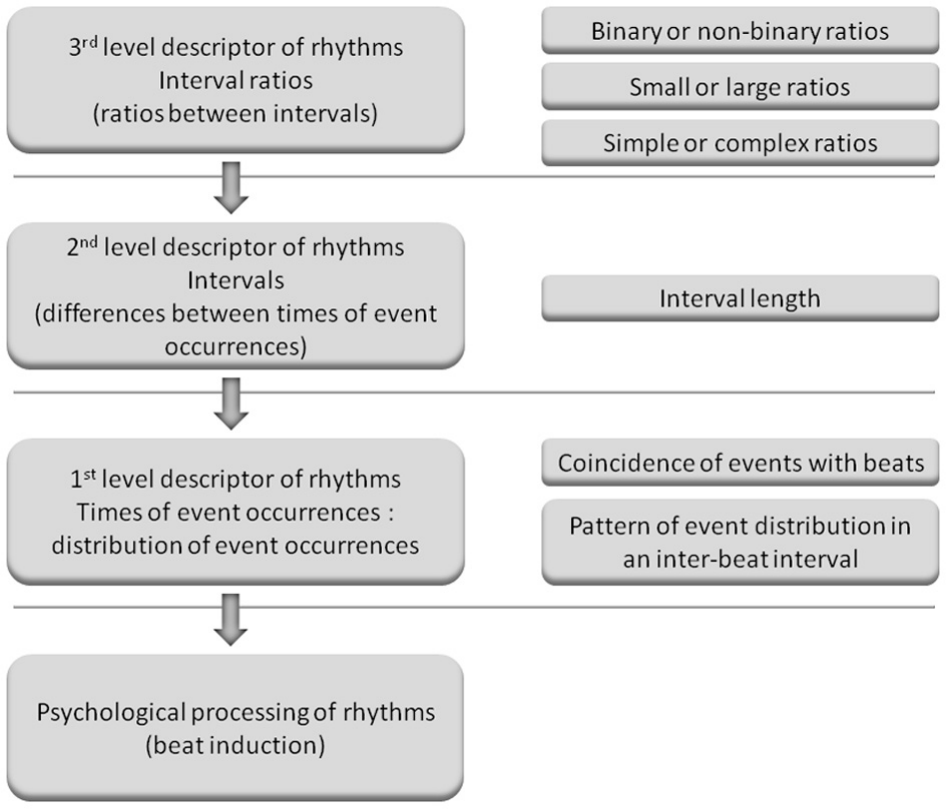
**Illustration of the three levels of descriptors of rhythms. (Left)** The possible relationships between different levels of descriptors of rhythms and psychological processing of rhythms. When listening to musical rhythms, the feeling of a beat is induced and beat induction is considered as psychological processing of rhythms. Musical rhythm refers to the organization of events in time in a hierarchical metrical structure, and therefore times of event occurrences may be most related to beat induction and are referred to as the first level descriptor. Intervals are derived from times of event occurrences and interval ratios are derived from intervals. Therefore, intervals and interval ratios are referred to as the second and third level descriptors, respectively. The third level descriptor may influence beat induction by the second level descriptor, and the second level descriptor may influence beat induction by the first level descriptor (as indicated by arrows). **(Right)** Measures for different levels of descriptors. For example, interval ratios can be examined in terms of magnitude, complexity, or type (binary or non-binary). Times of event occurrences can be represented as the distribution of event occurrences (event distribution), i.e., the numbers of events occurrences at different positions within a beat interval. The distribution of event occurrences can be measured by the coincidence of events with beats or the pattern of event distribution in a beat interval (event distribution pattern, as shown in the present study). (The concept of the three levels of descriptors was proposed in a personal communication with Bruno Repp. This figure is provided here to illustrate the logic behind the present study: after observing the binary advantage for serial interval ratios, further examining factors more related to psychological processing of rhythms. We have no intention to define a specific frame of the three levels of descriptors, e.g., influencing factors of rhythm processing can be classified into only three levels, or a given factor has to belongs to a given level).

It also remains to be elucidated how to more precisely evaluate metrical strength and predict rhythm task performance. Interval ratios may be a third level descriptor of rhythms (ratios between intervals); intervals may be a second level descriptor (differences between times of event occurrences); and times of event occurrences (distribution of event occurrences) may be a first level descriptor, which is plausibly most related to the metrical strength of rhythms (Figure 2). Therefore, if an advantage of serial binary over non-binary ratios could be observed in the present study, an important question to be further clarified is how serial binary ratios influence the distribution of event occurrences. Studies have investigated the distribution of event occurrences within a beat interval (inter-beat interval), with the emphasis on event occurrences at the beginning position in a beat interval, that is, the coincidence of events with beats (Povel and Essens, 1985; McAuley and Semple, 1999; Patel et al., 2005). Povel and Essens (1985) propose that people perceive and reproduce rhythms by structuring their representation according to an internal clock, which refers to internally generated periodic pulses (beats). If rhythms more match the interval clock, that is, rhythm events more coincide with the interval clock, the clocker-induction (beat induction) will be stronger. Experimental results show that strongly metrical rhythms have more events in coincidence with beats; they are better discriminated and reproduced than weakly metrical rhythms that have fewer events in coincidence with beats (Drake and Gérard, 1989; Patel et al., 2005; Chen et al., 2008; Grahn and Rowe, 2009).

If event occurrences at different positions within a beat interval were all taken into account, metrical strength may be better determined and rhythm performance may be better predicted. A frequency distribution method (Palmer and Krumhansl, 1990) is suitable for the purpose of analyzing event occurrences at different positions, and was adopted in the present study to investigate the distribution of event occurrences within a beat interval. The analysis method statistically covered all the possible positions of event occurrences in a beat interval by calculating the frequency with which an event occurred at a position. This analysis would emphasize the relationships between event occurrences at different positions, that is, a pattern of the distribution of event occurrences.

In addition, several studies have shown that rhythms with small integer ratios such as 2:1 or 3:1 are reproduced more accurately than rhythms with larger ratios such as 5:1, or complex non-integer ratios such as 2.5:1 or 3.5:1 (Essens, 1986; Sakai et al., 1999). These results suggest that the magnitude and complexity of interval ratios may also be factors influencing rhythm reproduction, which were also investigated in the current study.

The present study attempted to answer two questions: (1) whether rhythm reproduction could be more accurate with serial binary ratios than with serial non-binary ratios; (2) whether the distribution pattern of event occurrences within a beat interval could better predict rhythm reproduction performance, compared to the coincidence of events with beats, or the magnitude and complexity of ratios.

For the first purpose of the present study, an experimental paradigm that constructs rhythm sets with different ratio types and compares the reproduction of different rhythm sets (Sakai et al., 1999) was adopted, while with the following specific designs. (1) Rhythms formed with exclusively binary (1:2:4:8), non-binary integer (1:3:5:6) or non-integer (1:2.3:5.3:6.4) ratios were constructed. Binary ratios are necessarily integer ratios, whereas integer ratios are not necessarily binary ratios (e.g., ratios 1:3). To better distinguish the representation of binary ratios from that of integer ratios, the 1:3:5:6 rhythm type was the only rhythm type that used non-binary integer ratios. (2) All types of rhythm were constructed in a common 4/4 meter (which is the most common meter in Western music). Therefore, the effect of hierarchical ratios due to different meters was not involved. Another potential issue could be, different from non-binary ratios, binary ratios inevitably can be considered as hierarchical ratios due to a hierarchical structure with a 1:2 ratio between metrical levels. We emphasize that with the current design (different rhythm types in a common meter), the influence of the ratios was mainly the “serial” effect due to successive intervals in a rhythm; rather than the “hierarchical” effect due to the hierarchical structure underlying a rhythm (e.g., different meters). In other words, the present study mainly investigated the serial effect of the ratios in a rhythm and it would be appropriate to consider the binary ratios as serial ratios in a rhythm. As it has been suggested, “Hierarchical ratios are often used in music, but serial ratios are more commonly used in psychological studies of rhythm” (Repp et al., 2011). (3) The above-mentioned confounding factors (permutation of time intervals, number of time intervals, and rhythm duration) were all kept constant for different rhythm types. Each rhythm contained five intervals (with an immediate repetition of the shortest interval. This repetition of the shortest interval caused the sum of relative interval durations in a rhythm to be 16) and lasted for four beat intervals (a measure). A beat interval was 1200 ms in duration. The rhythms were produced by a systematic permutation of time intervals, with the same 24 permutations for each rhythm type (see the methods section for details).

For the second purpose of the present study, the frequency distribution method (Palmer and Krumhansl, 1990) was adopted to analyze the distribution of event occurrences within a beat interval.

In addition, external beats provide an explicit metrical reference for perception of rhythm (Essens and Povel, 1985; Grahn and Rowe, 2009) and were also investigated in the present study.

## Materials and methods

### Participants

Twelve subjects (five women; mean age ± *SD* 25.083 ± 5.534 years) participated in the main experiment. Three subjects were Americans and nine were Chinese. Ten subjects were right-handed and two were left-handed. Three Chinese subjects reported musical experience [one playing guitar for 10 years, one playing piano for 1 year, and one playing guzheng (a Chinese instrument) for 11 years]. Two right-handed Chinese subjects (one woman, age 28; one man, age 34. Without musical experience) participated in the control experiment. All subjects had normal hearing. The research protocols in this study were approved by the University of Minnesota or Sun Yat-Sen University. All subjects gave written, informed consents.

### Rhythms

Rhythms in 4/4 meter formed with binary 1:2:4:8, non-binary integer 1:3:5:6, or non-integer 1:2.3:5.3:6.4 ratios were produced. The subjects were informed of the meter and received practice in the pre-practice session. The purpose of the pre-practice session was to make the subjects understand the task and react according to the instruction (six keypresses for each rhythm, see below). In the main experiment, the beat interval was 1.2 s. While the most preferred beat interval is around 600 ms, the beat interval used in the main experiment is within the range of beat perception (Parncutt, 1994; Van Noorden and Moelants, 1999; Repp, 2006; Grahn and Brett, 2007). A control experiment with a 600 ms beat interval was also conducted. The control experiment was the same as the main experiment, except that the beat interval was 600 ms. In the main experiment, a rhythm lasted for four beat intervals (a measure, 4.8 s). The shortest base time interval (equal to a sixteenth note) was 300 ms in duration, which was the same for all rhythms and served as the reference for other intervals (intervals longer than the base interval were regarded as non-base intervals). The two base intervals were kept adjacent because shortest intervals often appear together in music. To generate different rhythms with the same ratio properties, the order of time intervals was systematically permuted, resulting in 24 rhythms for each rhythm type (see Table 1). The 24 permutations were the same for each rhythm type. The two base intervals were treated as one interval during permutation: e.g., permutation of four intervals in ratios 1:2:4:8 resulted in rhythms 1-1-2-4-8, 2-4-1-1-8, etc. The beginning of a rhythm was always marked by an event. The first 20 rhythms for each rhythm type in Table 1 were used in the formal experiment. The last four rhythms for each rhythm type in Table 1 (in the last row for each rhythm type) were used in the pre-practice session (including both condition one and condition two, see below) and were excluded from the analysis.

**Table 1:** **Rhythm list**.

**THE 1:2:4:8 RHYTHM TYPE**			
8-1-1-2-4	1-1-2-4-8	4-2-1-1-8	8-4-2-1-1
8-2-1-1-4	2-1-1-8-4	4-1-1-2-8	4-2-8-1-1
4-8-2-1-1	4-1-1-8-2	1-1-8-4-2	1-1-8-2-4
1-1-4-8-2	2-8-1-1-4	1-1-2-8-4	8-1-1-4-2
2-8-4-1-1	4-8-1-1-2	2-1-1-4-8	1-1-4-2-8
8-4-1-1-2	2-4-8-1-1	8-2-4-1-1	2-4-1-1-8
**THE 1:3:5:6 RHYTHM TYPE**			
6-1-1-3-5	1-1-3-5-6	5-3-1-1-6	6-5-3-1-1
6-3-1-1-5	3-1-1-6-5	5-1-1-3-6	5-3-6-1-1
5-6-3-1-1	5-1-1-6-3	1-1-6-5-3	1-1-6-3-5
1-1-5-6-3	3-6-1-1-5	1-1-3-6-5	6-1-1-5-3
3-6-5-1-1	5-6-1-1-3	3-1-1-5-6	1-1-5-3-6
6-5-1-1-3	3-5-6-1-1	6-3-5-1-1	3-5-1-1-6
**THE 1:2.3:5.3:6.4 RHYTHM TYPE**			
6.4-1-1-2.3-5.3	1-1-2.3-5.3-6.4	5.3-2.3-1-1-6.4	6.4-5.3-2.3-1-1
6.4-2.3-1-1-5.3	2.3-1-1-6.4-5.3	5.3-1-1-2.3-6.4	5.3-2.3-6.4-1-1
5.3-6.4-2.3-1-1	5.3-1-1-6.4-2.3	1-1-6.4-5.3-2.3	1-1-6.4-2.3-5.3
1-1-5.3-6.4-2.3	2.3-6.4-1-1-5.3	1-1-2.3-6.4-5.3	6.4-1-1-5.3-2.3
2.3-6.4-5.3-1-1	5.3-6.4-1-1-2.3	2.3-1-1-5.3-6.4	1-1-5.3-2.3-6.4
6.4-5.3-1-1-2.3	2.3-5.3-6.4-1-1	6.4-2.3-5.3-1-1	2.3-5.3-1-1-6.4

The numbers indicate relative interval durations, with 1 = 300 ms.

### Tasks

The sequence of rhythms in Table 1 was randomized for each type of rhythm, and the three types of rhythm were mixed randomly. This randomization procedure was performed once and the produced rhythm sequence was used for all subjects. During the experiment, a fixation point was permanently displayed on the center of a computer screen. The fixation point was a white disk on a black background and subtended a visual angle of 1.2°. The subjects were asked to fixate on the fixation point and to maintain attention on the task. A trial started with a 2 s written visual cue consisting of the word “Prepare” presented above the fixation point. Then the word in the cue was changed to “Listen” and a rhythm was successively presented three times (without silence between the three presentations) through a headphone monaurally. We attempted to simulate the real-life situation. When listening to music with a stereo earplug or headset, piano sounds (melody) are usually delivered to one ear, and drum sounds that mark the beginning of a measure (e.g., by large drum sounds) or a beat interval (e.g., by small drum sounds) are usually delivered to the other ear (see below for details). A whole presented sequence was 14.4 s. The rhythm was repeatedly presented to enhance rhythm perception (Povel and Essens, 1985; Grahn and Brett, 2007). The end of the final beat interval was marked (see below). After the rhythm presentation period, the word in the cue was changed to “Reproduce” and the subjects were asked to reproduce the rhythm one time by pressing a key on a computer keyboard (Grahn and Brett, 2007). It was emphasized that the end of the final interval of a rhythm had to be indicated by a keypress in the reproduction. Therefore, the reproduction of a rhythm included six keypresses marking the five reproduced intervals. The subjects had 9.6 s to reproduce the rhythm. The inter-trial interval was 2.8 s, indicated by the cue consisting of the word “Interval.” Whether the subjects pressed with their dominant hand was counterbalanced across subjects.

There were two conditions. In both conditions, the rhythm was composed of synthetic piano sounds (C5 on a piano keyboard), which were delivered to one ear (delivering to the left or right ear was counterbalanced across subjects). The sound of a large drum was delivered to the other ear (piano and drum sounds were delivered to different ears) on beat 1 of each rhythm cycle (the beginning of a rhythm), to help the subjects separate rhythm cycles (a rhythm was presented three times); and at the end of the final beat interval, to mark the end of stimulus presentation. The difference between the two conditions was that in the second one, the sound of a small drum was delivered to the ear to which the large drum sound was delivered on all four beats of each rhythm cycle (the sound of the large drum was still present. The sound of the large drum was at the beginning of each rhythm cycle, and the sound of the small drum was at the beginning of each beat interval), thus producing external beats. (The auditory examples are provided online. Audio 1–3: for the rhythms 1-1-2-4-8, 1-1-3-5-6, and 1-1-2.3-5.3-6.4 from condition one, respectively; Audio 4–6: for the rhythms 1-1-2-4-8, 1-1-3-5-6, and 1-1-2.3-5.3-6.4 from condition two, respectively). The sequence of the two conditions was counterbalanced across subjects. All sounds lasted for 100 ms. The drum sounds (large and small drum sounds) did not need to be reproduced.

### Data analyses

#### Frequency distribution

**Figure 3:**
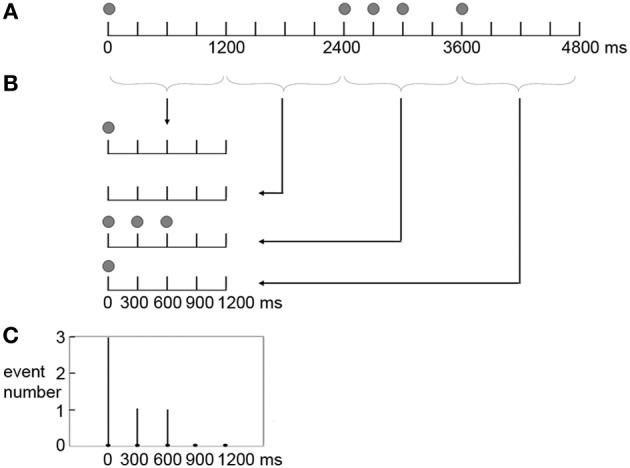
**Scheme of producing the frequency distribution of presented events in a beat interval.** Shown is the calculation for a sample rhythm 8-1-1-2-4. **(A)** The five events (indicated by filled circles) occur at 0, 2400, 2700, 3000, 3600 ms in a measure. **(B)** In terms of individual beat intervals, events occur at 0 ms in the 1st beat interval; 0, 300, and 600 ms in the 3rd beat interval; and 0 ms in the 4th beat interval. **(C)** Event occurrences in individual beat intervals are added up, resulting in 3 event occurrences at 0 ms, 1 event occurrence at 300 ms, and 1 event occurrence at 600 ms within a beat interval.

The frequency with which an event occurred at a position in a beat interval (the frequency distribution of presented events) was calculated for the three rhythm types. Each rhythm type had 20 distinct rhythms in the formal experiment. Take the rhythm 8-1-1-2-4 for example (Figure 3), the five events occurred at 0, 2400, 2700, 3000, 3600 ms in a measure. In terms of individual beat intervals, event positions were not the same in each of the four beat intervals. Events occurred at 0 ms in the 1st beat interval; 0, 300, and 600 ms in the 3rd beat interval; and 0 ms in the 4th beat interval. Event occurrences in individual beat intervals were then added up, resulting in 3 event occurrences at 0 ms, 1 event occurrence at 300 ms, and 1 event occurrence at 600 ms within a beat interval. This calculation was conducted for all 20 rhythms, and the numbers of event occurrences at different positions within a beat interval of all 20 rhythms were added up. After that, the numbers of event occurrences at different positions within a beat interval were transformed to percentages of the total number of event occurrences (100 event occurrences with 5 event occurrences in each of the 20 rhythms). The frequency distribution of presented events was calculated in the same way for the 1:3:5:6 and 1:2.3:5.3:6.4 rhythm types.

In addition, the frequency with which an event occurring at a position in a beat interval was incorrectly reproduced (the frequency distribution of incorrectly reproduced events) was also calculated for the three rhythm types. An incorrectly reproduced event was defined as the event starting an incorrectly reproduced interval (see the “order” section below for details about how incorrectly reproduced intervals were identified). The way to calculate the frequency distribution of incorrectly reproduced events was the same as the way to calculate the frequency distribution of presented events, except that the number of incorrectly reproduced events for each rhythm and the total number of incorrectly reproduced events depended on subjects' performance. Take the rhythm 8-1-1-2-4 for example: if the interval corresponding to 2 was reproduced longer than the interval corresponding to 4, these two intervals were regarded as incorrectly reproduced. The events starting the intervals corresponding to 2 and 4 occurred at 600 ms in the 3rd beat interval, and 0 ms in the 4th beat interval, respectively. The occurrences of incorrectly reproduced events in individual beat intervals were then added up, resulting in 1 occurrence at 0 ms, and 1 occurrence at 600 ms within a beat interval. This calculation was conducted for all 20 rhythms, and the numbers of occurrences of incorrectly reproduced events at different positions within a beat interval of all 20 rhythms were added up. After that, the numbers of the occurrences of incorrectly reproduced events at different positions within a beat interval were transformed to percentages of the total number of incorrectly reproduced events. The frequency distribution of incorrectly reproduced events was calculated in the same way for the 1:3:5:6 and 1:2.3:5.3:6.4 rhythm types.

#### Reproduction performance

Performance in the reproduction task was assessed using three measures: the order of intervals, the ratio of intervals, and the rhythm duration (Essens and Povel, 1985; Drake, 1993; Sakai et al., 1999; Grahn and Brett, 2007). The order was examined to evaluate whether a rhythm was correctly reproduced, while the ratio and duration were examined to obtain more precise information about reproduced rhythms (Sakai et al., 1999; Grahn and Brett, 2007).

For each rhythm, reproduction performance was analyzed based on the six keypresses marking the five reproduced intervals.

***Order***. The order of intervals in a rhythm refers to the sequence of relative durations of time intervals, which reflects the relative relationships between the durations of intervals. For example, in the rhythm 1-1-2-4-8, the two shortest intervals appeared first, followed by progressively longer intervals. If the reproduced order was different from the presented order, that is, if any reproduced interval was longer than another reproduced interval that was supposed to be longer (e.g., the interval corresponding to 2 was longer than the interval corresponding to 4) and vice versa (e.g., the interval corresponding to 4 was shorter than the interval corresponding to 2), a rhythm was regarded as being incorrectly reproduced. The incorrectly reproduced rhythms were not included in the following ratio and duration analyses.

To examine the influences of the magnitude and complexity of interval ratios on reproduction performance (see Incorrectly Reproduced Intervals in the Results and discussion section) and the distribution pattern for subjects' performance (see Frequency Distribution of Incorrectly Reproduced Events in the Results and discussion section), analyses were also conducted to identify incorrectly reproduced intervals in an incorrectly reproduced rhythm. There could be two or more incorrectly reproduced intervals in an incorrectly reproduced rhythm. For the rhythm 1-1-2-4-8, if the reproduced fifth interval was shorter than the reproduced fourth interval, intervals corresponding to 4 and 8 were both regarded as being incorrectly reproduced; if the reproduced fifth interval was shorter than the reproduced third interval, intervals corresponding to 2, 4, and 8 were all regarded as being incorrectly reproduced, etc. In addition, for the two base intervals corresponding to 1, if the duration difference between the two reproduced base intervals was greater than the duration difference between the shorter reproduced base interval and the reproduced interval corresponding to 2, two base intervals and the interval corresponding to 2 were all regarded as being incorrectly reproduced.

***Ratio***. The reproduced non-base intervals were divided by the mean of the two base intervals. The ideal ratios would be 2:1, 4:1, and 8:1 for the 1:2:4:8 rhythm type; 3:1, 5:1, and 6:1 for the 1:3:5:6 rhythm type; and 2.3:1, 5.3:1, and 6.4:1 for the 1:2.3:5.3:6.4 rhythm type.

***Duration***. The duration of the reproduced rhythm was compared to the duration of the presented rhythm, which was 4.8 s for all rhythms.

The three measures were independent and complementary to each other, together providing comprehensive information about reproduced rhythms. Take the rhythm 8-1-1-2-4 for example, if the interval corresponding to 2 was reproduced longer than the interval corresponding to 4, the rhythm would be reproduced closer to another rhythm 8-1-1-4-2. Therefore, the order was examined to evaluate whether a rhythm was correctly reproduced. The interval corresponding to 2 could be reproduced longer than the length that it was supposed to be, but still shorter than the interval corresponding to 4. In this case, the rhythm would still be reproduced as 8-1-1-2-4 (i.e., the same rhythm), though not accurately, as reflected by the ratio. Furthermore, the rhythm could be reproduced with the correct order and accurate ratios, but may be faster or slower than the presented tempo, as reflected by the rhythm duration. Therefore, the ratio and rhythm duration were examined to obtain more precise information about reproduced rhythms.

#### Statistics

The Greenhouse–Geisser corrections were applied to all analysis of variance (ANOVA) analyses. Bonferroni corrections were applied to all *t*-tests and corrected *p*-values <0.05 were considered significant. All *t*-tests were two-tailed.

## Results and discussion

### Frequency distribution of presented events

**Figure 4:**
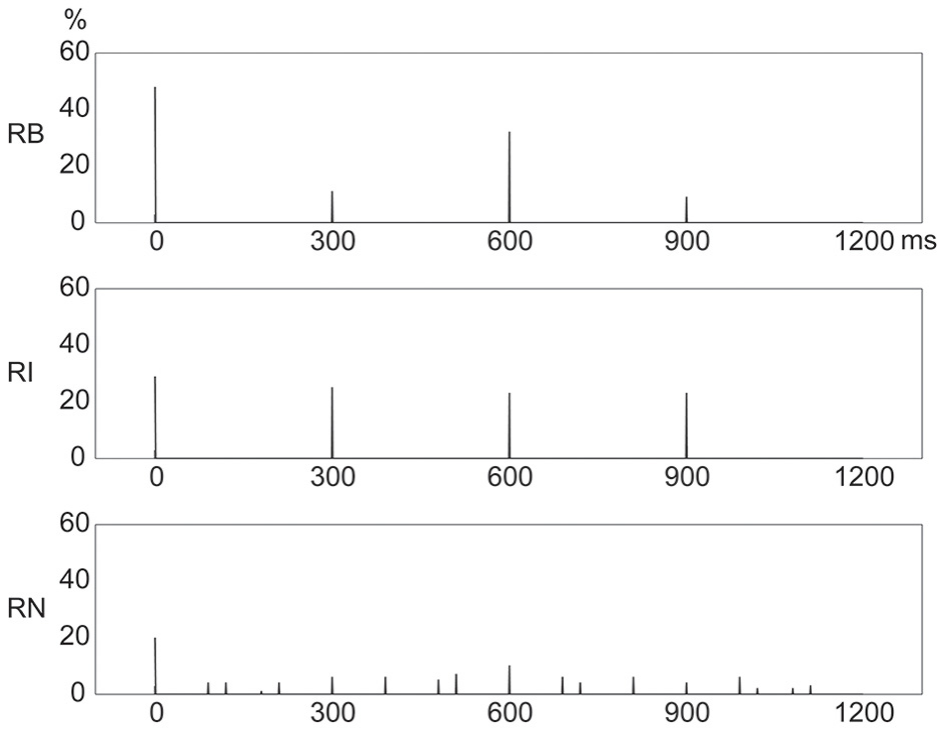
**Frequency distributions of presented events in a beat interval.** The three types of rhythm had distinct frequency distributions of rhythm events. *Y* axis: percent of event occurrences. *X* axis: positions with event occurrences in a beat interval 1200 ms in duration. RB, RI, and RN represent rhythms formed with binary, non-binary integer, and non-integer ratios, respectively.

Figure 4 shows distinct frequency distributions of presented events for the three rhythm types. For the 1:2:4:8 rhythm type, most events occurred at the beginning (48%) and 1/2 (32%) positions within a beat interval, and few events occurred at the 1/4 (11%) and 3/4 (9%) positions. For the 1:3:5:6 rhythm type, the events were almost evenly distributed between the beginning (29%), 1/4 (25%), 1/2 (23%), and 3/4 (23%) positions. For the 1:2.3:5.3:6.4 rhythm type, events were widely distributed, with 20% at the beginning and 10% at the 1/2 position.

Given the coincidence of events with beats, these results would predict that reproduction performance would be best for the 1:2:4:8 rhythm type because of the large number of event occurrences at the beginning position in a beat interval; and that reproduction performance would be worst for the 1:2.3:5.3:6.4 rhythm type due to the small number of event occurrences at the beginning position and the wide distribution of event occurrences throughout a beat interval (Drake and Gérard, 1989; Patel et al., 2005; Chen et al., 2008; Grahn and Rowe, 2009).

**Figure 5:**
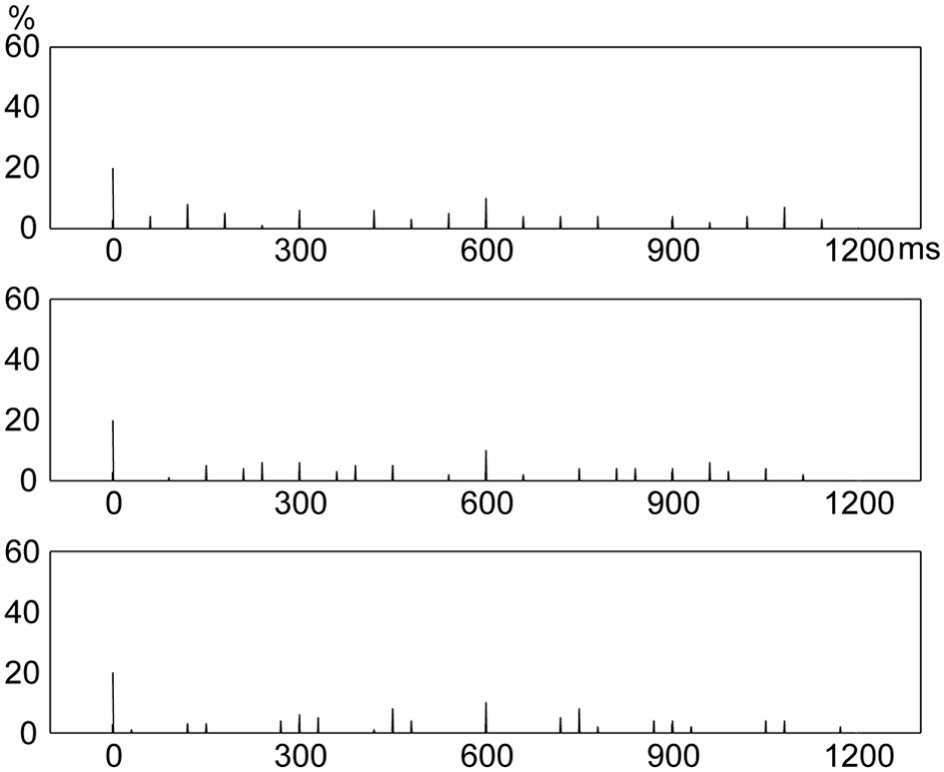
**Frequency distributions of presented events in a beat interval for three additional groups of non-integer ratios.** Rhythms formed with non-integer ratios 1:1, 2.2:1, 4.4:1, and 7.4:1 **(Top)**; 1:1, 2.7:1, 4.8:1, and 6.5:1 **(Middle)**; and 1:1, 1.6:1, 5.5:1, and 6.9:1 **(Bottom)** all resulted in frequency distributions of presented events similar to that for rhythms formed with ratios 1:1, 2.3:1, 5.3:1, and 6.4:1 (see Figure 4, bottom). *Y* axis: percent of event occurrences. *X* axis: positions with event occurrences in a beat interval 1200 ms in duration.

In the current study, the relative duration of an interval was less than or equal to 8:1 (relative to the base interval). A rhythm contained five intervals and the sum of relative interval durations was 16 (with the repetition of the base interval). Rhythms with binary ratios consisted of ratios 1:1, 2:1, 4:1, and 8:1; and rhythms with non-binary integer ratios consisted of ratios 1:1, 3:1, 5:1, and 6:1. However, rhythms with non-integer ratios could consist of ratios other than 1:1, 2.3:1, 5.3:1, and 6.4:1. Therefore, one concern may be that rhythms with other non-integer ratios (e.g., 1:1, 2.2:1, 4.4:1, and 7.4:1) might result in frequency distributions different from the frequency distribution of rhythms with ratios 1:1, 2.3:1, 5.3:1, and 6.4:1. This issue was addressed by examining frequency distributions of presented events for rhythms formed with three additional groups of randomly generated non-integer ratios (1:2.2:4.4:7.4, 1:2.7:4.8:6.5, or 1:1.6:5.5:6.9). The method to construct rhythms (see Rhythms in the Materials and Methods section) and the method to produce the frequency distribution of presented events (see the first paragraph in Frequency Distribution in the Materials and Methods section) were the same for the three additional groups of non-integer ratios and the 1:2.3:5.3:6.4 ratios. The results showed that rhythms with the three additional groups of non-integer ratios all resulted in frequency distributions of event occurrences (Figure 5) similar to that for the 1:2.3:5.3:6.4 rhythm type (Figure 4, bottom).

### Reproduction performance

Rhythms with incorrect numbers of keypresses were excluded from the analysis. In the 20 rhythms for each rhythm type in the formal experiment, there were 1.250 ± 1.138 (mean ± *SD*), 0.667 ± 0.778, and 1.083 ± 1.240 rhythms with incorrect numbers of keypresses for the 1:2:4:8, 1:3:5:6, and 1:2.3:5.3:6.4 rhythm types, respectively, in condition one; and 1.250 ± 0.965, 1.167 ± 1.337, and 1.333 ± 0.985 rhythms with incorrect numbers of keypresses for the 1:2:4:8, 1:3:5:6, and 1:2.3:5.3:6.4 rhythm types, respectively, in condition two. A Two-Way repeated measures ANOVA, with the factors external beats (without external beats in condition one, with external beats in condition two) and rhythm types (three rhythm types), showed no significant main effect or interaction.

#### Order

**Figure 6:**
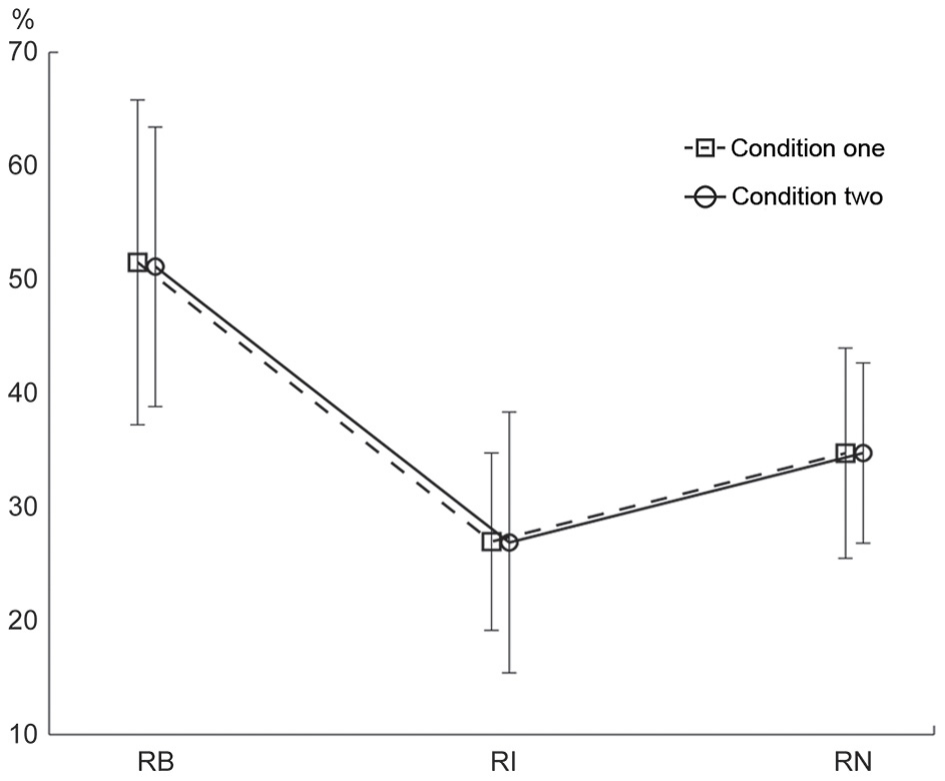
**Percentage of correctly reproduced rhythms.** The percentage of correctly reproduced rhythms was higher for the 1:2:4:8 rhythm type than for the 1:3:5:6 and 1:2.3:5.3:6.4 rhythm types. Error bars indicate ±95% confidence intervals (Loftus and Masson, 1994; Masson and Loftus, 2003). RB, RI, and RN represent rhythms formed with binary, non-binary integer, and non-integer ratios, respectively.

The percentage of correctly reproduced rhythms is illustrated in Figure 6. A Two-Way repeated measures ANOVA, with the factors external beats (without external beats in condition one, with external beats in condition two) and rhythm types (three rhythm types), showed a significant main effect for rhythm types [*F*_(2, 22)_= 46.173, *p* < 0.001, partial η^2^ = 0.808]. Because there was no significant interaction between external beats and rhythm types, the data from the two conditions were combined in *post-hoc* analyses. The results showed that the percentage of correctly reproduced rhythms was higher for the 1:2:4:8 rhythm type than for the 1:3:5:6 rhythm type [*t*_(11)_ = 8.473, *p* < 0.001, η^2^ = 0.867] and the 1:2.3:5.3:6.4 rhythm type [*t*_(11)_ = 5.636, *p* < 0.001, η^2^ = 0.743]. In addition, the percentage of correctly reproduced rhythms was higher for the 1:2.3:5.3:6.4 rhythm type than for the 1:3:5:6 rhythm type [*t*_(11)_ = 4.355, *p* = 0.001, η^2^ = 0.633]. For the three subjects with musical experience, the differences in order performance between three rhythm types were also observed (the percentage of correctly reproduced rhythms was 65.388 ± 20.940, 36.158 ± 18.960, and 49.203 ± 17.205 for the 1:2:4:8, 1:3:5:6, and 1:2.3:5.3:6.4 rhythm types, respectively), although in general their order performance was better than the order performance from all subjects (as shown in Figure 6) (because there were only three subjects with musical experience, a direct statistical comparison between order performance from the subjects with or without musical experience was not conducted).

The sequence of the two conditions was counterbalanced. One concern is whether the differences in order performance between three rhythm types were influenced by condition sequences. This issue was addressed by a Three-Way repeated measures ANOVA, with the factors condition sequences (received condition one first or received condition two first. Between-subjects effect), external beats (without external beats in condition one, with external beats in condition two) and rhythm types (three rhythm types). Same as the above Two-Way ANOVA, a significant main effect for rhythm types [*F*_(2, 20)_ = 44.220, *p* < 0.001, partial η ^2^ = 0.816] was observed. The results also showed a significant interaction between condition sequences and external beats [*F*_(1, 10)_= 12.299, *p* = 0.006, partial η^2^ = 0.552], suggesting a general learning or training effect: the performance was better for the condition conducted first than for the condition conducted afterward. Other main effects and other interactions were not significant. Importantly, the interaction between condition sequences and rhythm types was not significant [*F*_(2, 20)_ = 0.535, *p* = 0.547, partial η^2^ = 0.051], suggesting that the differences in order performance between three rhythm types were not influenced by condition sequences.

Whether the subjects pressed with their dominant hand was counterbalanced across subjects. In order to test whether the assignment of the dominant hand would influence the differences in order performance between three rhythm types, a Three-Way repeated measures ANOVA, with the factors dominant hand (using the dominant hand or not. Between-subjects effect), external beats (without external beats in condition one, with external beats in condition two) and rhythm types (three rhythm types), was conducted. Same as the above Two-Way ANOVA, a significant main effect for rhythm types [*F*_(2, 20)_ = 46.659, *p* < 0.001, partial η^2^ = 0.824] was observed. Other main effects and other interactions were not significant. Importantly, the interaction between dominant hand and rhythm types was not significant [*F*_(2, 20)_ = 1.116, *p* = 0.347, partial η^2^ = 0.100], suggesting that the differences in order performance between three rhythm types were not influenced by the assignment of the dominant hand.

Better order performance for the 1:2:4:8 rhythm type than for the 1:3:5:6 and 1:2.3:5.3:6.4 rhythm types is consistent with the prediction implied by the number of event occurrences at the beginning position in a beat interval. The 1:2.3:5.3:6.4 rhythm type had the fewest events coinciding with beats and had a wide event distribution, but the order performance for the 1:2.3:5.3:6.4 rhythm type was better than that for the 1:3:5:6 rhythm type. This is contrary to the prediction implied by the number of event occurrences at the beginning position. Therefore, the coincidence of events with beats alone cannot explain the order results. The order results could be better interpreted by the distribution pattern of event occurrences. Both 1:2:4:8 and 1:2.3:5.3:6.4 rhythm types showed a distribution pattern with more events at the beginning than at any other positions in a beat interval, whereas the 1:3:5:6 rhythm type showed an almost even distribution pattern. Hence, although the 1:3:5:6 rhythm type had more events at the beginning position than the 1:2.3:5.3:6.4 rhythm type had, the order performance for the 1:3:5:6 rhythm type was the worst. These results suggest that reproduction performance may be better predicted by the distribution pattern of event occurrences within a beat interval, than by the coincidence of events with beats alone.

In the 20 rhythms for each rhythm type in the formal experiment, there were 9.083 ± 4.274, 14.083 ± 2.234, and 12.333 ± 2.807 incorrectly reproduced rhythms for the 1:2:4:8, 1:3:5:6, and 1:2.3:5.3:6.4 rhythm types, respectively, in condition one; and 9.167 ± 3.762, 13.833 ± 3.857, and 12.167 ± 2.480 incorrectly reproduced rhythms for the 1:2:4:8, 1:3:5:6, and 1:2.3:5.3:6.4 rhythm types, respectively, in condition two. These incorrectly reproduced rhythms were not included in the following ratio and duration analyses.

**Figure 7:**
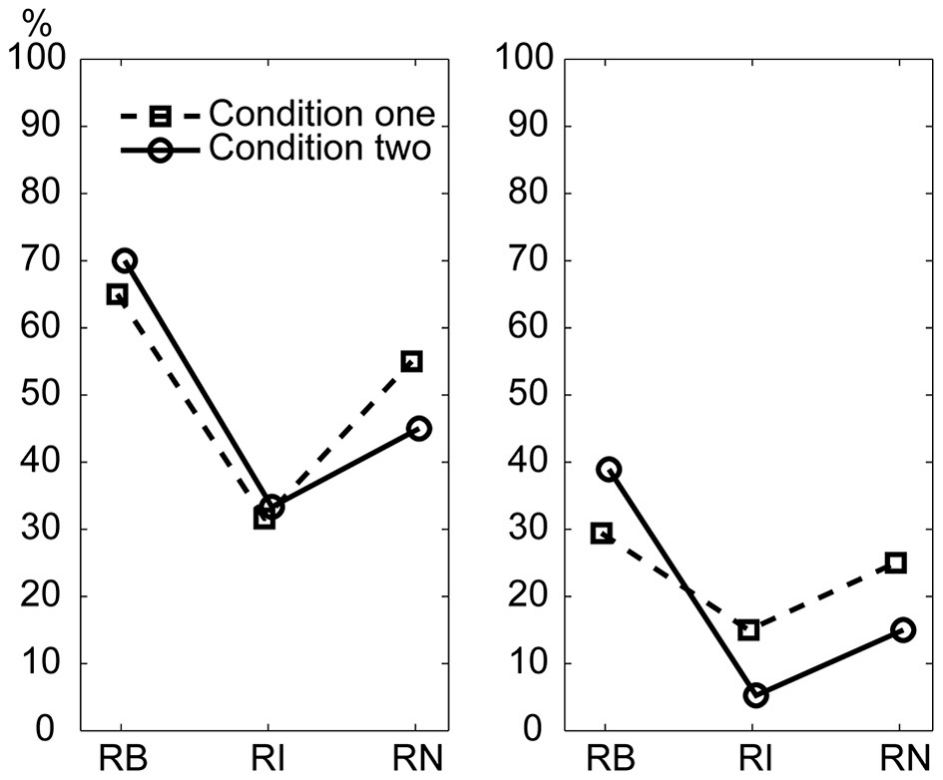
**Percentage of correctly reproduced rhythms with a 600 ms beat interval. Left:** subject 1; **Right:** subject 2. RB, RI, and RN represent rhythms formed with *b*inary, non-binary *i*nteger, and *n*on-integer ratios, respectively.

A control experiment was conducted to test whether the superiority of serial binary over non-binary ratios observed in the main experiment could also be observed when a 600 ms beat interval was used. The percentage of correctly reproduced rhythms is illustrated in Figure 7. For both of the two subjects in the control experiment, the percentage of correctly reproduced rhythms was higher for the 1:2:4:8 rhythm type than for the 1:3:5:6 rhythm type and the 1:2.3:5.3:6.4 rhythm type; and the percentage of correctly reproduced rhythms was higher for the 1:2.3:5.3:6.4 rhythm type than for the 1:3:5:6 rhythm type. These results are consistent with the results of the main experiment.

The current criterion that defined whether a rhythm was correctly reproduced may be strict and the order performance (Figure 6) was poor. Here we tested a “less strict” criterion (Drake and Palmer, 2000; Chen et al., 2008; Brown et al., 2012) that assesses global rhythm accuracy (GRA) by the percentage of correctly reproduced intervals in a rhythm, with an interval being considered incorrectly reproduced if the reproduced interval differs from the present interval by more than 50% (Drake and Palmer, 2000). The results showed that GRA was 80.942 ± 8.397%, 87.651 ± 5.812%, and 82.489 ± 7.090% for the 1:2:4:8, 1:3:5:6, and 1:2.3:5.3:6.4 rhythm types, respectively, in condition one; and 80.916 ± 8.584%, 85.843 ± 4.941%, and 80.819 ± 6.147% for the 1:2:4:8, 1:3:5:6, and 1:2.3:5.3:6.4 rhythm types, respectively, in condition two. A Two-Way repeated measures ANOVA, with the factors external beats (without external beats in condition one, with external beats in condition two) and rhythm types (three rhythm types), showed a significant main effect for rhythm types [*F*_(2, 22)_= 8.297, *p* = 0.010, partial η^2^ = 0.430]. Because there was no significant interaction between external beats and rhythm types, the data from the two conditions were combined in *post-hoc* analyses. The results showed that GRA was higher for the 1:3:5:6 rhythm type than for the 1:2:4:8 rhythm type [*t*_(11)_= 2.777, *p* = 0.018, η^2^ = 0.412] and the 1:2.3:5.3:6.4 rhythm type [*t*_(11)_= 3.812, *p* = 0.003, η^2^ = 0.569]. The measure of GRA that examines reproduction accuracy by a window of time may be more related to the measure of ratio used in the present study (see the ratio results below, small deviation of the reproduced ratio from the presented ratio was observed for the 1:3:5:6 rhythm type). Importantly, the GRA results and the order results are not conflicting, because different measures provide different information about the reproduced rhythms. As it is stated, GRA “globally informs us whether subjects knew the sequence, but critically, is not informative about the timing of each action within the sequence” (Chen et al., 2008). Therefore, the GRA results and the order results are supplementary to each other, supporting the validity of the present data.

#### Ratio

**Figure 8:**
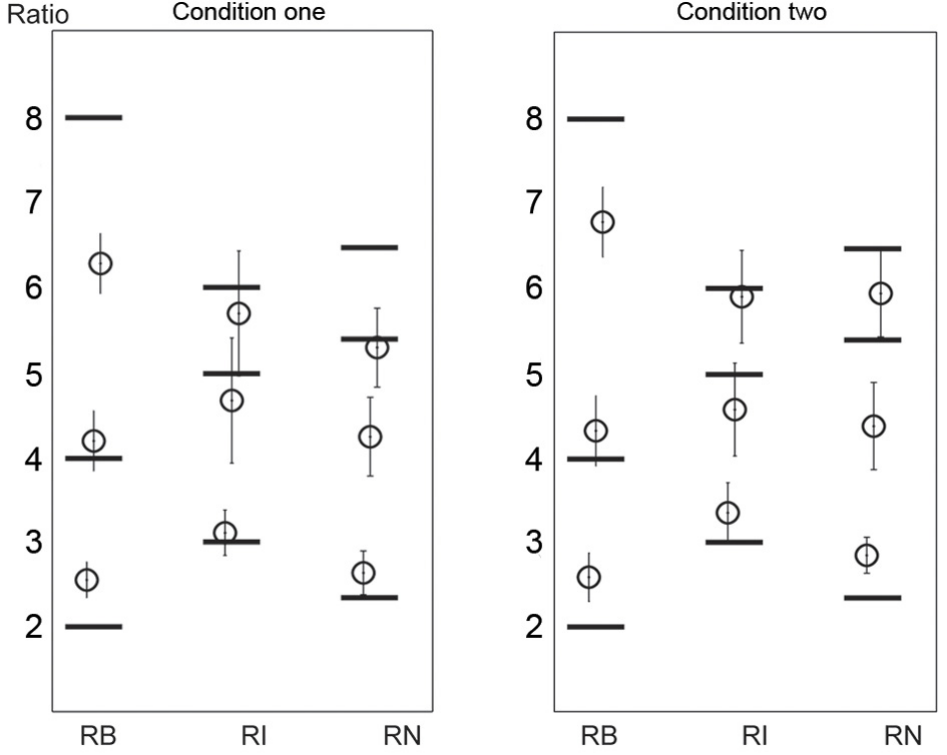
**Reproduced ratios.** Horizontal black lines mark the presented ratios. Error bars indicate ±95% confidence intervals. RB, RI, and RN represent rhythms formed with *b*inary, non-binary *i*nteger, and *n*on-integer ratios, respectively.

The ratio analyses (and the following duration analyses) were performed on the correctly reproduced rhythms (as indicated in Figure 6). The reproduced ratios are shown in Figure 8. Because the pattern of the results was similar in the two conditions, the data from the two conditions were combined. For the 1:2:4:8 rhythm type, the reproduced ratio for 2:1 was larger than 2:1 [*t*_(11)_ = 5.861, *p* < 0.001, η^2^ = 0.757], and that for 8:1 was smaller than 8:1 [*t*_(11)_ = 3.640, *p* = 0.004, η^2^= 0.546]. For the 1:2.3:5.3:6.4 rhythm type, the reproduced ratio for 2.3:1 was larger than 2.3:1 [*t*_(11)_ = 4.925, *p* < 0.001, η^2^ = 0.688], and that for 5.3:1 was smaller than 5.3:1 [*t*_(11)_ = 5.060, *p* < 0.001, η^2^ = 0.700]. (The comparison between reproduced and presented ratios was conducted on all ratios for each rhythm type. No other significant differences were found apart from those reported above). There seemed to be a relationship between the reproduced order and ratio: the more accurate the reproduced order, the less accurate the reproduced ratio. But this relationship was weak (the significant deviation of the reproduced ratio from the presented ratio was only observed on two ratios for the 1:2:4:8 or the 1:2.3:5.3:6.4 rhythm type). This issue may be addressed in future studies.

#### Duration

The duration of the reproduced rhythm was 4.446 ± 0.360 s, 4.652 ± 0.545 s, and 4.368 ± 0.386 s for the 1:2:4:8, 1:3:5:6, and 1:2.3:5.3:6.4 rhythm types, respectively, in condition one; and 4.495 ± 0.644 s, 4.518 ± 0.608 s, and 4.537 ± 0.574 s for the 1:2:4:8, 1:3:5:6, and 1:2.3:5.3:6.4 rhythm types, respectively, in condition two. The reproduced rhythm duration was significantly shorter than the presented (4.8 s) rhythm duration for the 1:2:4:8 [*t*_(11)_ = 3.403, *p* = 0.006, η^2^ = 0.513] and the 1:2.3:5.3:6.4 [*t*_(11)_ = 3.885, *p* = 0.003, η^2^ = 0.578] rhythm types in condition one. This may be partly related to the large deviations of the reproduced ratios from the presented ratios for the 1:2:4:8 and 1:2.3:5.3:6.4 rhythm types.

### Incorrectly reproduced intervals

To test whether the magnitude and complexity of interval ratios were primary factors influencing the current results, incorrectly reproduced intervals were analyzed. The incorrectly reproduced intervals were classified into the base interval, the 1st non-base interval, the 2nd non-base interval, and the 3rd non-base interval according to the length of intervals for each rhythm type. If the magnitude and complexity of interval ratios were major factors influencing the performance in the present reproduction task, there would be two predictions about the results. The first was regarding the magnitude of ratios: the error rate would increase from the base interval to the 3rd non-base interval (corresponding to small to large ratios). The second was regarding the complexity of ratios: non-base intervals would be worst reproduced for the 1:2.3:5.3:6.4 rhythm type (corresponding to complex ratios relative to the base interval).

**Figure 9:**
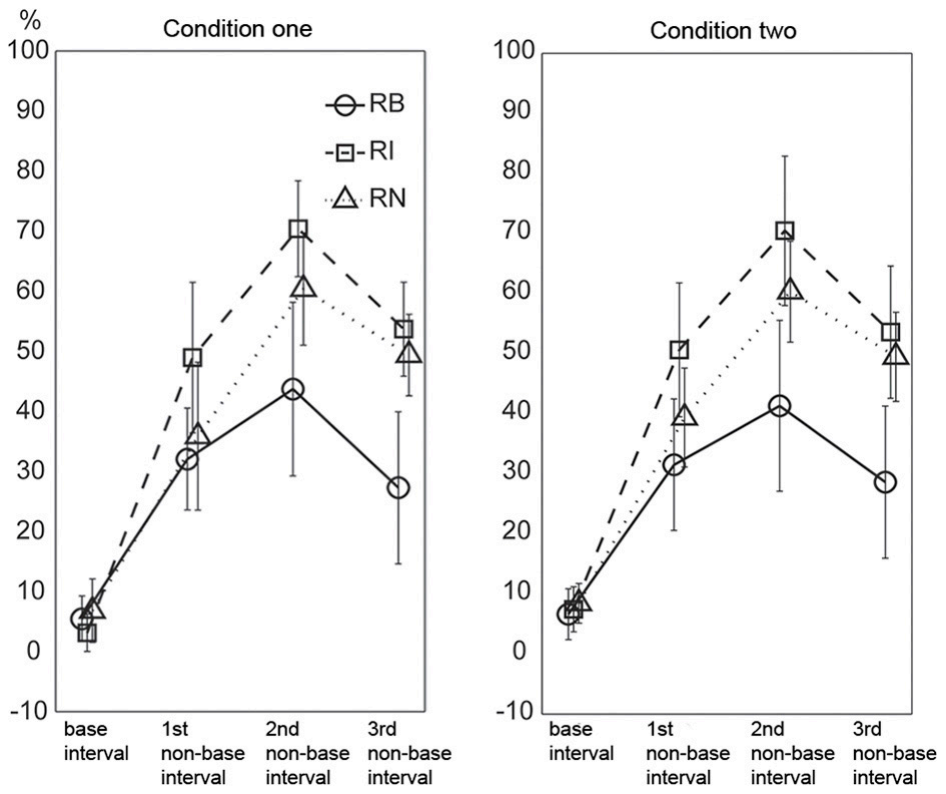
**Percentage of incorrectly reproduced intervals.** For non-base intervals, the reproduced error rate was lower for the 1:2:4:8 rhythm type than for the 1:3:5:6 and 1:2.3:5.3:6.4 rhythm types. Error bars indicate ±95% confidence intervals. RB, RI, and RN represent rhythms formed with *b*inary, non-binary *i*nteger, and *n*on-integer ratios, respectively.

The results are illustrated in Figure 9. A Three-Way repeated measures ANOVA, with the factors external beats (without external beats in condition one, with external beats in condition two), rhythm types (three rhythm types), and interval durations (the base interval, the 1st, 2nd, and 3rd non-base intervals), showed significant main effects for rhythm types [*F*_(2, 22)_= 42.100, *p* < 0.001, partial η^2^ = 0.793] and interval durations [*F*_(3, 33)_= 149.396, *p* < 0.001, partial η^2^ = 0.931], and a significant interaction between rhythm types and interval durations [*F*_(6, 66)_ = 17.661, *p* < 0.001, partial η^2^ = 0.616].

The two predictions based on the magnitude and complexity of interval ratios were not observed in the results. First, the results did not show a general trend of error rates increasing from the base interval to the 3rd non-base interval. Although the error rate of the base interval was low, the error rate was not higher for the 3rd non-base interval than for the 1st and 2nd non-base intervals (inconsistent with the first prediction). Second, the error rates of non-base intervals were highest for the 1:3:5:6 rhythm type, rather than for the 1:2.3:5.3:6.4 rhythm type (inconsistent with the second prediction).

The high error rate of the 2nd non-base interval may be partly due to the calculation method identifying incorrectly reproduced intervals (at least two intervals involved). But the influence of the calculation method should be almost equal for the 1st and 3rd non-base intervals. The error rate of the base interval was low (6.033 ± 5.169, 5.267 ± 4.043, and 7.604 ± 5.781 percents for the three rhythm types, respectively). If only non-base intervals were taken into account, the 1st and 3rd non-base intervals should be almost equally influenced by the calculation method. Therefore, at least for the 1st and 3rd non-base intervals, the result (the error rate was not higher for the 3rd than for the 1st non-base interval) may not be due to the influence of the calculation method. We also did an extreme test. The error rate of the base interval was added to the error rate of the 3rd non-base interval, and then compared to the error rate of the 1st non-base interval (data from the two conditions were combined). For the three rhythm types, only the comparison for the 1:2.3:5.3:6.4 rhythm type was significant [*t*_(11)_ = 7.398, *p* < 0.001, η^2^ = 0.817].

There could be another concern regarding the ratios between intervals rather than the ratio of an interval to the base interval. For example, the ratio between the 2nd and 3rd non-base intervals in the 1:2:4:8 rhythm type (4:8) was larger than that in the 1:3:5:6 rhythm type (5:6), and thus, the 2nd and 3rd non-base intervals would be expected to be easier to discriminate in the 1:2:4:8 rhythm type than in the 1:3:5:6 rhythm type in the present study. Ratio differences for the three types of ratios (1:2:4:8, 1:3:5:6, and 1:2.3:5.3:6.4) were 1:2:4, 2:2:1, and 1.3:3:1.1, respectively; and their sums were 7:1, 5:1, and 5.4:1, respectively. This seemed to explain why the 1:2:4:8 rhythm type was more accurately reproduced than the other two rhythm types. However, the reproduction performance of individual intervals (Figure 9) could not be explained. Ratio differences for the three ratio types showed increased (1:2:4), decreased (2:2:1), and reversed-U (1:3.3:1.1) patterns, respectively, whereas the performance of individual intervals showed the reversed-U pattern for all three ratio types. More specifically, the 2nd and 3rd non-base intervals were not easier to discriminate in the 1:2:4:8 rhythm type (in a 4:8 ratio) than in the 1:3:5:6 rhythm type (in a 5:6 ratio) (as shown in Figure 9, the error rate difference between the 2nd and 3rd non-base intervals was similar for the 1:2:4:8 and 1:3:5:6 rhythm types).

In addition, the error rates of non-base intervals were lower for the 1:2:4:8 rhythm type than for the 1:3:5:6 and 1:2.3:5.3:6.4 rhythm types, and lower for 1:2.3:5.3:6.4 rhythm type than for the 1:3:5:6 rhythm type. These results measuring the accuracy of an individual interval were consistent with the results measuring the accuracy of a whole rhythm (see above “order” analyses).

These results may not support the notion that the magnitude and complexity of interval ratios could be primary factors influencing the performance of the present reproduction task.

### Frequency distribution of incorrectly reproduced events

There may also be a distribution pattern for subjects' performance, that is, the relationships between the chances with which reproduction errors occurred at different positions within a beat interval. Theories of rhythm perception emphasize events occurring at the beginning position within a beat interval (Povel and Essens, 1985; Patel et al., 2005), and it is suggested that such events would attract more attention (Jones and Boltz, 1989; Large and Jones, 1999) and an error would thus be less likely to occur at the beginning than at other positions. A different view could be, the chance with which an error occurs at a position is largely determined by the chance with which an event is presented at that position. This view concerns the relationships between event occurrences at different positions within a beat interval, that is, the distribution pattern of event occurrences. One way to differentiate the two hypotheses may be to compare the frequency distribution of incorrectly reproduced events with the frequency distribution of presented events. Particularly, if the chance of event occurrence during presentation is the same at all positions, the two views would give different predictions regarding the chance of error occurrence in performance. The chance of error occurrence would not be the same at all positions (with less chance at the beginning position) according to the view emphasizing the beginning position; and would be the same at all positions according to the view emphasizing the chance of event occurrence. The 1:3:5:6 rhythm type had almost equal numbers of presented events at individual positions, therefore providing a near ideal example to test the two views. In other words, the view emphasizing the beginning position would not predict a high similarity between frequency distributions of presented and incorrectly reproduced events, especially for the 1:3:5:6 rhythm type.

**Figure 10:**
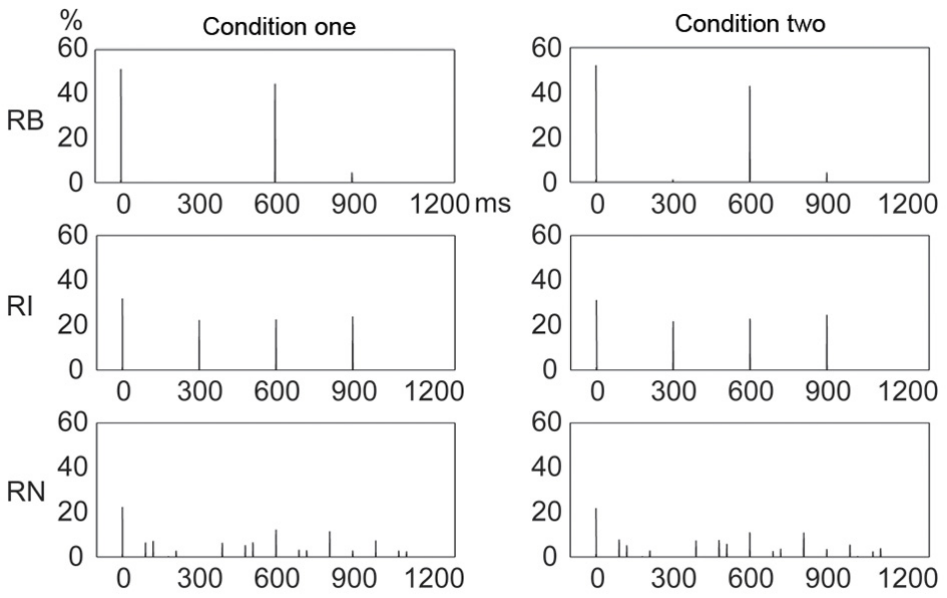
**Frequency distributions of incorrectly reproduced events in a beat interval.** The frequency distribution of incorrectly reproduced events was highly similar to that of presented events (Figure 4) for all rhythm types in both conditions. Y axis: percent of occurrences of incorrectly reproduced events. *X* axis: positions with the occurrence of incorrectly reproduced events in a beat interval 1200 ms in duration. RB, RI, and RN represent rhythms formed with binary, non-binary integer, and non-integer ratios, respectively.

Figure 10 illustrates the frequency distribution of incorrectly reproduced events, which showed high similarity, under visual inspection, to the frequency distribution of presented events (Figure 4). This observation was confirmed by further correlation analyses between frequency distributions of incorrectly reproduced and presented events, with *r*^2^> 0.872 for all rhythm types in both conditions. Particularly, for the 1:3:5:6 rhythm type, an almost even frequency distribution of presented events resulted in an almost even frequency distribution of incorrectly reproduced events. These results unlikely support the view emphasizing the beginning position.

## General discussion

The present study investigated the reproduction of rhythms formed with binary, non-binary integer, and non-integer ratios. The results showed that rhythms with binary ratios were more accurately reproduced than rhythms with non-binary ratios, suggesting a superiority of serial binary over non-binary ratios in rhythm reproduction. The results further showed that (1) rhythms with non-integer ratios were more accurately reproduced than rhythms with non-binary integer ratios; (2) the magnitude and complexity of ratios were not major factors influencing the current reproduction performance; (3) the chance with which an error occurred at a position within a beat interval was largely determined by the chance with which an event was presented at that position. (See the above Results and discussion section for a detailed discussion). These results suggest that the distribution pattern of event occurrences within a beat interval may better determine metrical strength and better predict reproduction performance, compared to the coincidence of events with beats, or the magnitude and complexity of ratios. Serial binary ratios may result in strong metrical strength (indicated by e.g., a distribution pattern with more events at the beginning than at any other positions), and by which improve reproduction performance.

The meter is an internal construct and listeners may not perceive it as intended (Povel and Essens, 1985; Patel et al., 2005; Repp et al., 2008). A potential issue of the present study is whether the subjects perceived 4/4 meter as intended. For three reasons, we think that the subjects did perceive 4/4 meter. First, the subjects were informed of the intended meter and received practice in the pre-practice session. Second, the results from the two conditions of the present study (without or with an external beat) showed similar results. Third, two subjects did a control experiment with a 600 ms beat interval and showed results consistent with the results of the main experiment having a 1200 ms beat interval, which indicates that the subjects perceived the intended 4/4 meter with both 600 and 1200 ms beat intervals.

In the present study, the subjects were informed of the 4/4 meter. Therefore, beat induction was not totally spontaneous, but rather was influenced by the given meter. The introduction of external beats was to strength the induction of the intended beat. In other words, the difference between the two conditions without or with external beats was not spontaneous vs. non-spontaneous beat induction; but rather was weak vs. strong induction of the intended beat. But the effect of external beats was not observed in the present results, as reflected by the lack of performance difference between the two conditions. This issue remains to be addressed in future studies.

It should be emphasized that the present results did not rule out the influence of the coincidence of events with beats, or the magnitude and complexity of ratios on reproduction performance. For example, the better order performance for the 1:2:4:8 rhythm type than for the 1:2.3:5.3:6.4 rhythm type may be partly because the former had more events at the beginning position in a beat interval than the latter. Generally, the performance of the current reproduction task (see Figure 6) was poor (may partly depending on the measure used to assess the performance, as indicated by the GRA results), which may be partly due to the large and complex ratios involved in the task. What we emphasize is that the coincidence of events with beats or the magnitude and complexity of ratios, alone, cannot interpret the present results; whereas the current results could be better interpreted by the distribution pattern of event occurrences within a beat interval. In addition, the advantage for hierarchical binary over non-binary ratios in rhythm perception and reproduction (Rena, 1987; Drake, 1993; Bergeson and Trehub, 2006; Gerry et al., 2010) may be largely culture-dependent and may not be found in some cultures. For instance, Turkish infants, who are familiar with both Western meters and 7/8 Balkan meters, do not prefer duple over Balkan meter as Western infants do (Soley and Hannon, 2010). Therefore, whether the advantage for serial binary over non-binary ratios observed in the present reproduction task is culture-dependent remains to be examined in future studies.

## Conclusion

Rhythm theories and empirical data emphasize the role of the coincidence of events with beats in determining metrical strength and predicting rhythm performance. The present results suggest that rhythm processing may be better understood when the distribution pattern of event occurrences is taken into account. Future research would further examine how rhythm performance could be precisely predicted, e.g., by establishing a formal model encompassing the distribution pattern of event occurrences.

### Conflict of interest statement

The authors declare that the research was conducted in the absence of any commercial or financial relationships that could be construed as a potential conflict of interest.
